# Visualization of the hepatic and renal cell uptake and trafficking of tetrahedral DNA origami in tumour

**DOI:** 10.1111/cpr.13643

**Published:** 2024-04-04

**Authors:** Shitai Zhu, Hongzhen Peng, Huating Kong, Qinglong Yan, Kai Xia, Lihua Wang, Ying Zhu, Shihua Luo

**Affiliations:** ^1^ Division of Physical Biology, CAS Key Laboratory of Interfacial Physics and Technology Shanghai Institute of Applied Physics, Chinese Academy of Sciences Shanghai China; ^2^ University of Chinese Academy of Sciences Beijing China; ^3^ Shanghai Synchrotron Radiation Facility, Shanghai Advanced Research Institute, Chinese Academy of Sciences Shanghai China; ^4^ Institute of Materiobiology, College of Sciences, Shanghai University Shanghai China; ^5^ Xiangfu Laboratory Jiashan China; ^6^ Shanghai Frontier Innovation Research Institute Shanghai China; ^7^ Department of Traumatology Rui Jin Hospital, School of Medicine, Shanghai Jiao Tong University Shanghai China

## Abstract

DNA nanostructures, known for their programmability, ease of modification, and favourable biocompatibility, have gained widespread application in the biomedical field. Among them, Tetrahedral DNA Origami (TDOs), as a novel DNA nanostructure, possesses well‐defined structures, multiple modification sites, and large cavities, making it a promising drug carrier. However, current understanding of TDOs' interactions with biological systems, particularly with target cells and organs, remains unexplored, limiting its further applications in biomedicine. In this work, we prepared TDOs with an average particle size of 40 nm and labelled them with Cy5 fluorescent molecules. Following intravenous injection in mice, the uptake of TDOs by different types of liver and kidney cells was observed. Results indicated that TDOs accumulate in renal tubules and are metabolized by Kupffer cells, epithelial cells, and hepatocytes in the liver. Additionally, in a tumour‐bearing mouse model, TDOs passively targeted tumour tissues and exhibited excellent tumour penetration and retention after rapid metabolism in hepatocytes. Our findings provide crucial insights for the development of TDO‐based drug delivery systems.

## Graphical abstract

After intravenous injection in mice, tetrahedral DNA origami (TDOs) accumulate in renal tubules and are metabolized by Kupffer cells, epithelial cells, and hepatocytes in the liver. In a tumour‐bearing mouse model, TDOs passively target tumour tissues and exhibit excellent tumour penetration and retention after rapid metabolism in hepatocytes.

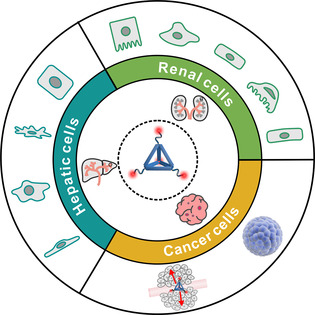

## 1 INTRODUCTION

DNA nanostructures have garnered significant attention in the biomedical field due to their remarkable abilities for precise molecular‐scale construction.^1^ Utilizing the principles of base pair complementarity, a variety of DNA nanostructures with well‐defined geometries,^2^, ^3^, ^4^, ^5^ excellent biocompatibility,^1^ precise programmability,^6^, ^7^, ^8^ and modifiability^9^, ^10^, ^11^ have been constructed. These structures have demonstrated broad potential applications in biological imaging,^12^, ^13^, ^14^, ^15^, ^16^ drug delivery,^17^, ^18^, ^19^ and disease diagnostics and treatment.^20^, ^21^ In particular, the tetrahedral DNA nanostructures (TDNs), known for entering cells via a ‘corner‐attack’ mechanism,^22^ have shown notable advantages as drug delivery carriers due to their small angles which provide more contact points and stronger interactions, thus leading to increased cellular uptake efficiency.^23^ However, the loading capacity of TDNs is limited due to the small size of the tetrahedral cavity; specifically, the central cavity of TDNs could accommodate a sphere with a radius of approximately 2.6 nm.^24^

DNA origami, formed by assembling a long scaffold strand with multiple short staple strands into diverse geometric shapes,^25^ such as triangles and squares,^26^, ^27^, ^28^, ^29^, ^30^, ^31^ has been widely applied in biomedicine,^32^, ^33^ due to its ability to carry large amounts of drugs owing to its large and complex structure among DNA nanostructures. Tetrahedral DNA Origami (TDOs) is a type of tetrahedral‐shaped DNA origami structure.^34^ Each edge of TDOs is composed of a 10‐helix bundle with a length of around 40 nm. TDOs possess precisely defined structures and multiple modification sites,^35^ which facilitate their abundant cellular uptake via a ‘corner‐attack’ mechanism, similar to TDNs. Several DNA nanostructures with varied sizes and shapes^23^, ^36^, ^37^, ^38^, ^39^ have been reported to enter cells via scavenger receptor‐mediated pathways. Given the similar properties of TDOs to above DNA nanostructures, such as its size, geometry, and surface properties, it is hypothesized that TDOs similarly utilize scavenger receptor‐mediated mechanisms for cellular internalization. Furthermore, due to its larger internal cavity estimated to accommodate a sphere with a radius of approximately 15.3 nm,^24^ TDOs are expected to exhibit superior drug loading capabilities compared to both TDNs and two‐dimensional DNA origami,^40^ thus holding promise as a new generation of DNA‐based nanoscale drug delivery systems. However, to date, TDOs have predominantly been employed as templates for assembling highly ordered nanoparticle superlattices,^34^, ^41^ while their interactions within biological systems and targeting of organs and cells remaining unreported.

**FIGURE 1:**
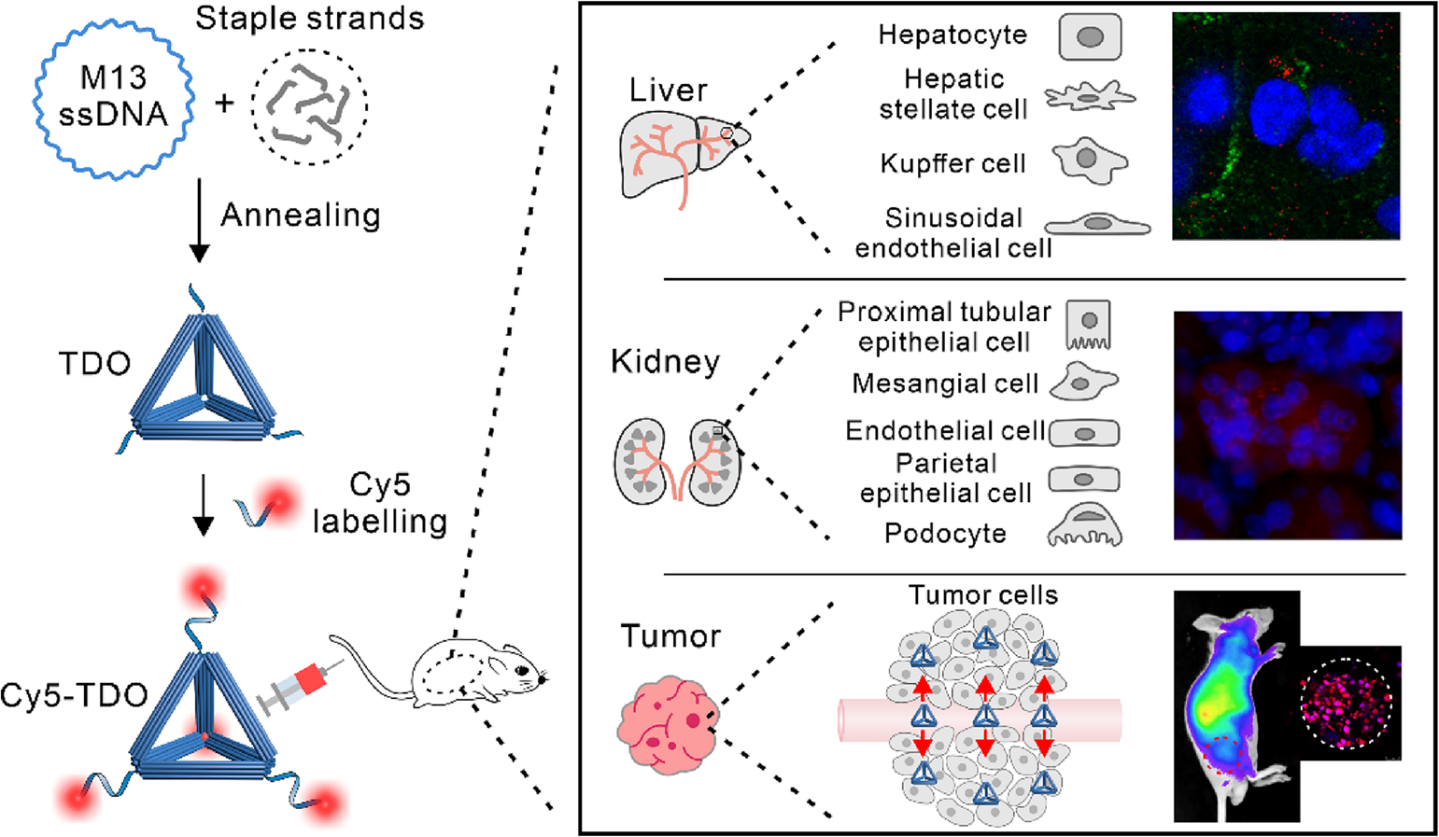
Experimental scheme. Cy5‐TDOs were prepared and their interactions with various target organs and cells within mice were systematically investigated after intravenous injection. Furthermore, the tumour‐targeting capacity of TDOs was assessed in tumour‐bearing mice and 3D spheroid tumour model.

In this study, TDOs with a diameter of around 40 nm were prepared, and their interactions with various target organs and cells within mice were systematically investigated following intravenous injection. Additionally, the tumour‐targeting capability of TDOs was systematically evaluated in tumour‐bearing mice and 3D spheroid tumour model, with the exploration of potential mechanisms involved (Figure 1).

## 2 MATERIALS AND METHODS

### 2.1 Materials

Chemicals and reagents were obtained from Sigma‐Aldrich unless stated otherwise. RPMI‐1640 containing L‐glutamine, Trypsin–EDTA (0.05%), fetal bovine serum (FBS) and penicillin–streptomycin (PS; 10 kU/mL) were obtained from Gibco. All oligonucleotides were purchased from Sangon (Shanghai, China). The staple strands were resuspended and diluted in water to a final concentration of 100 μmol/L.

### 2.2 Production of M13 bacteriophage ssDNA

The extraction of m13mp18 viral DNA was based on Shih et al.'s methods.^42^ Briefly, *Escherichia coli* was infected with the filamentous bacteriophages with M13 circular genome. Then the bacteriophage replicated to produce progeny phages that extrude directly into the culture medium without causing bacterial lysis. Following this phage expansion step, the cells were centrifuged and the phages were precipitated from the supernatant with 4% PEG‐8000/3% NaCl. DNA and protein in progeny phages were separated by alkaline denaturation and acid renaturation. The ssDNA genome was extracted and purified to be used as a source of ssDNA. Finally, the M13 ssDNA was resuspended in 10 mmol/L Tris (pH 8.5), and the concentration and quality of M13 ssDNA were characterized by UV–visible spectroscopy and a 1% agarose gel, respectively.

### 2.3 Cy5‐TDOs preparation and characterization

TDOs were assembled according to Rothemund's method. The molar ratio of the M13 ssDNA (10 nmol/L) and staple strands (100 nmol/L) was 1:10. The TDOs were assembled in 1 × TE/Mg^2+^ buffer (40 mmol/L Tris, 2 mmol/L EDTA and 12.5 mmol/L magnesium chloride, pH 8.0) by slowly cooling from 95 to 16°C through PCR program over 20 h. Concentration and purification of TDOs were achieved by PEG purification. To label TDOs with Cy5, several complementary Cy5‐labelled strands was incubated with prepared TDOs for overnight. The molar amount of Cy5‐labelled ssDNA was 10 times that of the molar amount of labelling sites to insure that each site was conjugated with Cy5 molecules.^43^ The diameter and zeta potential of TDOs were analysed by a zetasizer nano‐ZS90 particle analyser (Malvern). AFM imaging was conducted in Scan Analyst‐air mode (Multi‐mode 8, Bruker). 5 μL sample was spotted onto freshly cleaved mica and left for adsorption with 10 mmol/L NiCl_2_ for 5 min. Then it was observed by the microscope. For TEM imaging, 5 μL sample was pipetted onto the carbon‐coated copper grid. After deposition for 5 min, excess sample solution was removed with a piece of filter paper, and then 5 μL 1% uranyl acetate solution was dropped for negative staining for 1 min. Next, the grid was washed by ddH_2_O and dried in air, before being analysed by TEM (Tecnai G2 F20, FEI).

### 2.4 Cell culture

HepG2 cells were cultured in RPMI‐1640 complete medium (containing 10% FBS, 100 U/mL penicillin and 100 U/mL streptomycin) in 5% CO_2_ atmosphere at 37°C.

### 2.5 Animal experiments

BALB/c nude mice (male, 18–22 g) were purchased from Shanghai SLAC Laboratory Animal Co. Ltd., China. BALB/c nude mice were kept in pathogen‐free conditions (18–22°C, 50%–70% relative humidity, 12 h light–dark cycle). Permission of the local ethics committee was obtained, and all animal experiments were performed according to Chinese law and accepted international standards in biomedical research.

In the in vivo distribution experiments, BALB/c nude mice were anaesthetised and tail‐vein injected with 300 nmol Cy5‐TDOs. The mice were administered with 300 nmol Cy5‐TDOs via tail vein injection. At 0, 0.1, 1, 3, 6, 12 and 24 h post‐injection, the mice were imaged by animal optical imaging system (Berthold). The fluorescence light intensity of liver and kidney was analysed and normalized as counts per centimetre squared (cts/mm^2^).

In the tumour‐targeting experiments, 2 × 10^6^ HepG2 cells in 200 μL NS were subcutaneously injected in the right hind limb of nude mice to form the tumour‐bearing mice. When the tumour size reached 100 mm^3^ or so, the mice were administered with 300 nmol Cy5‐TDOs via tail vein injection. At 0, 0.1, 0.5, 1, 3 and 6 h post‐injection, the mice were imaged by animal optical imaging system (Berthold). At 0.5 h post‐injection, several mice were sacrificed with major organs harvested for ex vivo imaging. The fluorescence light intensity of tumour, heart, liver, spleen, lung and kidney was analysed and normalized as counts per centimetre squared (cts/mm^2^).

In the in vivo biocompatibility assessment experiment, BALB/c nude mice were tail‐vein injected with 300 nmol Cy5‐TDOs. At 0, 1, 6 and 24 h post‐injection, blood samples were collected and analysed.

### 2.6 Confocal imaging analysis

The liver and kidney were excised from the mice intravenously administrated with Cy5‐TDOs at 1 h post‐injection. The freshly dissected tissue was embedded, frozen and sectioned to prepare frozen‐section slides. For estimation of the location of Cy5‐TDOs in liver, the frozen‐section slides were stained with Kupffer cells (F4/80, 1:200), hepatocyte (albumin, 1:200), Hepatic stellate cell (Desmin, 1:200) and Sinusoidal endothelial cells (CD‐31, 1:200). Followed by Alexa 488‐labelled secondary antibody treatment, the slides were stained with Hoechst 33258. For estimation of the location of Cy5‐TDOs in kidneys, the frozen‐section slides were stained with Hoechst 33258 immediately. The slides were then mounted and imaged by confocal microscopy (Leica SP8).

### 2.7 Hepatotoxicity and nephrotoxicity analysis

Serum levels of ALT and AST activities, TP, Crea, BUN and UA concentrations were determined according to the detection kit (Nanjing Jiancheng Bioengineering Institute, China). The results were expressed as U/L, U/L, g/L, g/L, g/L, g/L and μmol/L, respectively.

### 2.8 Elisa analysis

Serum levels of TNF‐α, IFN‐α and IL‐6 were analysed by the corresponding ELISA kits (Pepro Tech) with 6 independent measurements (6 mice) of each cytokine.

### 2.9 Histopathological analysis

Paraffin‐embedded heart, liver, spleen, lung and kidney sections of mice were stained with haematoxylin and eosin and examined by optical microscopy. The pathologist performing the visual analysis was blind to the grouping of mice.

### 2.10 Confocal microscopy of Cy5‐TDOs treated 3D tumour cell models

The 3D multicellular tumour spheroids (MTSs) were applied to evaluate the penetrating ability of Cy5‐TDOs in vitro. Briefly, HepG2 cells were seeded on the agarose‐coated plates and cultured for 3 days to obtain MTSs. When it grew to ~100 μm in diameter, Cy5‐TDOs were co‐cultured with the MTSs for 4 h and collected, fixed and stained by Hoechst 33258 for confocal microscopy (Leica SP8) with Z‐stacking scanning.

### 2.11 Statistical analysis

All results are expressed as the mean ± standard deviation from triplicate experiments performed in a parallel manner. Statistical significance of the data was determined by *t*‐tests or one‐way analysis of variance (ANOVA) using SPSS. *p < 0.05; **p < 0.01; ***p < 0.001.

## 3 RESULTS

### 3.1 Assembly and characterization of TDOs

**FIGURE 2:**
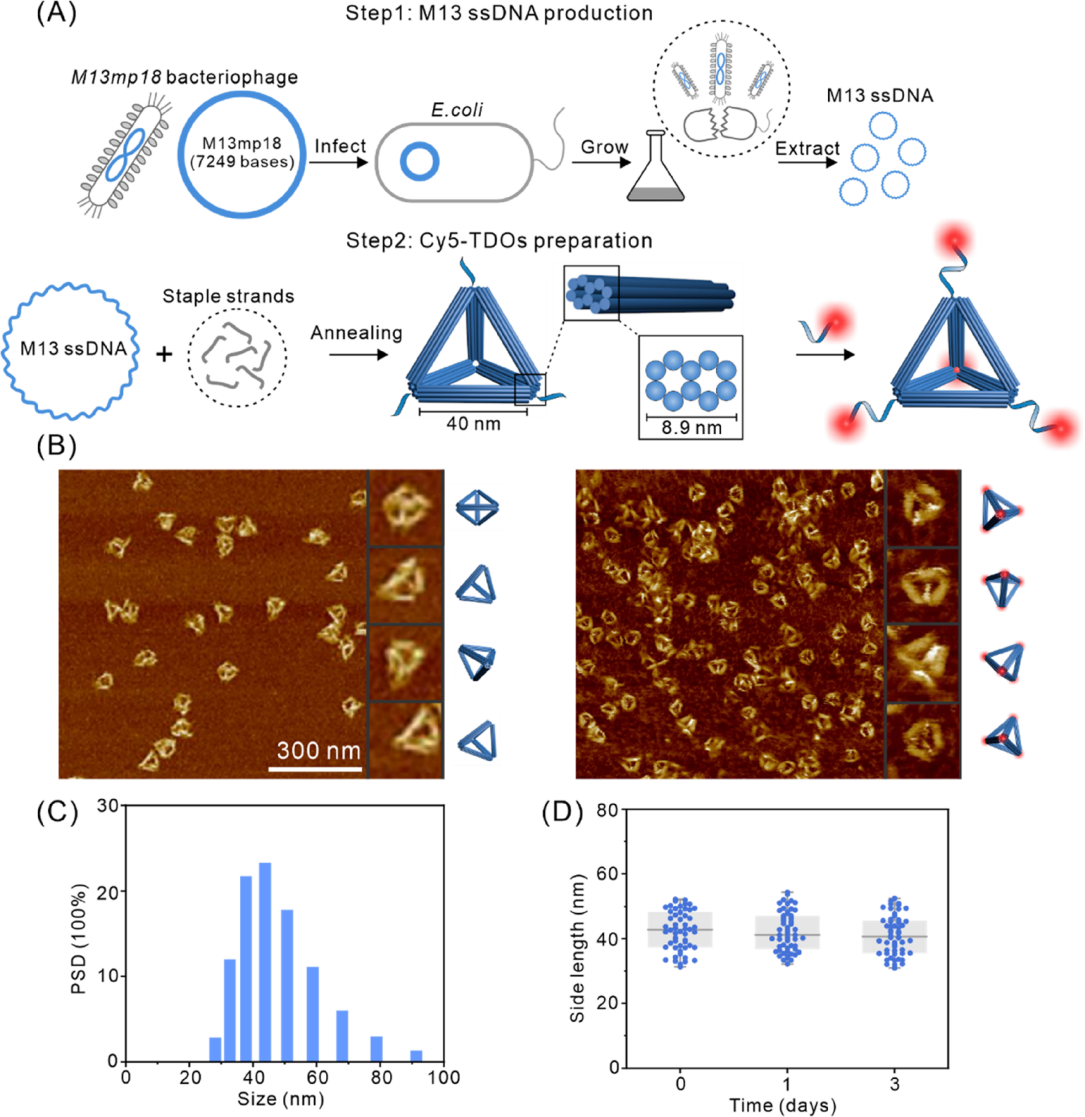
Cy5‐TDOs preparation. (A) Schematic illustration of the extraction of the M13 scaffold ssDNA and assembly of Cy5‐TDOs. (B) AFM images of TDOs before and after Cy5 labelling (scale bar: 300 nm). (C) DLS measurement statistics of TDOs. PSD: particle size distribution. (D) Statistical analysis of edge lengths of TDOs stored in TE/Mg^2+^ buffer over 0, 1, and 3 days.

The TDOs were assembled using the conventional single‐step annealing method. Initially, we expanded the culture of *E.coli* cells and infecting them with bacteriophages carrying a modified 7249‐base M13 genome, followed by bacteriophage lysis, collection, and purification of the 7249‐base M13 single‐stranded DNA (ssDNA) for subsequent experiments (Figure 2A).^42^ The purified 7249‐base M13 ssDNA was characterized using agarose gel electrophoresis. The results showed a clear single band, confirming the successful purification and correct length of the ssDNA (Figure S1). Subsequently, the 7249‐base M13 scaffold ssDNA were mixed with staple strands (Table S1) and assembled into the TDOs through a single annealing step. Theoretically, the TDOs consists of six rigid 10‐helix bundles, each approximately 40 nm in length with a cross‐sectional diameter of about 8.9 nm (Figure 2A). The AFM and TEM imaging confirmed the nanoscale morphology of the TDOs, exhibiting a well‐defined and monodisperse nanostructure (Figures 2B and S2). Cy5‐labelled ssDNA was attached to the vertices of the TDOs through base complementary pairing, with six modification sites per vertex (Table S2), resulting in a total of 24 Cy5 molecules per TDO (Figure 2A). AFM characterization of the Cy5‐TDOs revealed no significant differences with TDOs (Figure 2B), indicating that the fluorescent tagging did not affect the TDOs' structure. DLS analysis revealed hydrated diameters of 45.3 ± 2.4 and 46.1 ± 4.3 nm for the TDOs and Cy5‐TDOs, respectively (Figures 2C and S3). Zeta potential measurements indicated a zeta potential of −5.31 mV for the TDOs (Figure S4), due to the negative charge carried by DNA. The edge lengths of the TDOs stored in TE/Mg^2+^ buffer for 0, 1, and 3 days were observed and statistically analysed using AFM, revealing no significant structural changes and stable edge lengths around 45 nm, demonstrating the TDOs' stability in TE/Mg^2+^ buffer (Figures 2D and S5). We also assessed their stability in RPMI 1640 cell culture medium containing 10% FBS (Figure S6). It was observed that partial integrity of TDOs was retained after 24 h, indicating that TDOs possess some degree of stability in physiological conditions.

### 3.2 Visualization of the hepatic and renal cell uptake of TDOs

**FIGURE 3:**
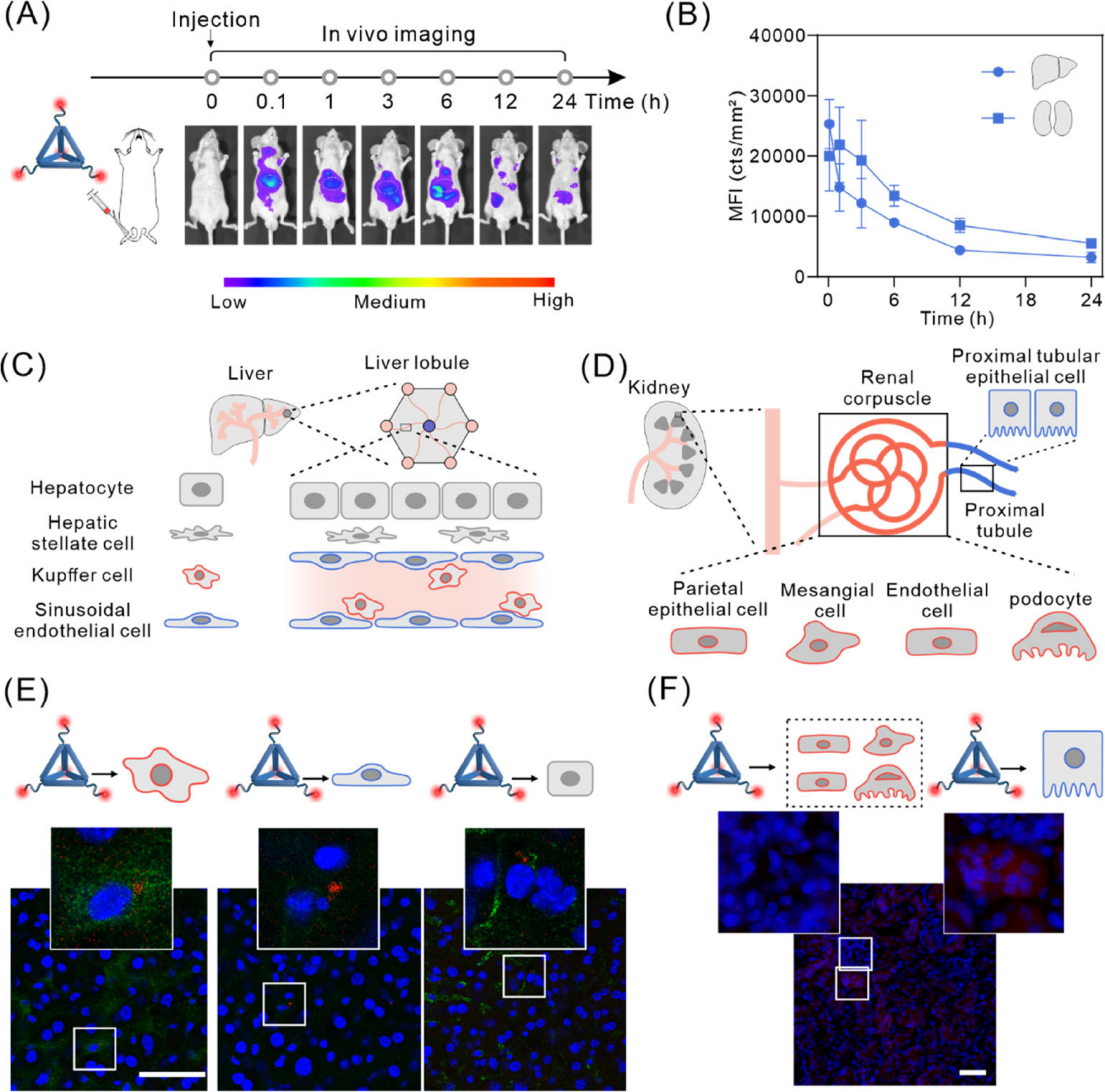
Visualization of the hepatic and renal cell uptake of TDOs. (A) Time‐dependent whole‐body images of mice after tail vein administration with Cy5‐TDOs. (B) Quantification of TDOs accumulated in liver and kidneys at different time points after tail vein administration with Cy5‐TDOs (*n* = 4, mean ± standard deviation). (C, D) Schematic illustrations of liver and kidney cells. (E, F) The distribution of Cy5‐TDOs in various hepatic and renal cell types was examined, utilizing Hoechst 33258 for nuclear staining, F4/80 to mark Kupffer cells, CD‐31 for endothelial cell labelling, and albumin staining for hepatocyte identification. Primary antibodies were visualized using secondary antibodies tagged with green fluorescence, scale bar: 50 μm. Quantitative analysis showed mean fluorescence intensities of Cy5‐TDOs in Kupffer cells, endothelial cells, and hepatocytes as 1.04 ± 0.29, 0.84 ± 0.53, and 0.61 ± 0.31, respectively.

Next, the distribution of TDOs in various target organs and cells after systemic administration was investigated. The in vivo distribution of TDOs was determined by imaging mice at 0, 0.1, 1, 3, 6, 12, and 24 h post intravenous injection of Cy5‐TDOs. The results revealed that fluorescence signal primarily accumulated in the liver and kidneys. Notably, the fluorescence intensity peaked at 0.1 h in the liver and gradually decreased thereafter. In contrast, in the kidneys, the peak intensity was observed at 1 h followed by a gradual decline (Figure 3A). Quantitative analysis of the fluorescence intensity in liver and kidneys (Figure 3B) corresponded with the imaging results, indicating that TDOs predominantly distributed in the liver and kidneys. Similar to tetrahedral framework nucleic acids (tFNAs)^44^ and rectangular DNA origami nanostructures (Rec‐DON),^45^ the fluorescence intensity in the liver rapidly declined within 1 h, halving between 0.1 and 1 h. In contrast to tFNAs and Rec‐DON, the fluorescence intensity in the liver at 0.1 h post intravenous injection of TDOs was significantly higher than that in the kidneys, possibly due to the steric hindrance of the 40 nm size of TDOs, which might prevent it from crossing the glomerular capillary walls and resulting in preferential accumulation in the liver.^46^ Regarding the changes in fluorescence within the kidneys, in difference with the rapid metabolism of tFNAs directly in the kidneys, both TDOs and Rec‐DON showed an initial increase followed by a subsequent decrease within 3 h, suggesting possible accumulation of TDOs in the renal tubules. To further explore the cellular distribution of TDOs in the liver and kidneys, cryosections were prepared 1‐h post injection of TDOs, and subjected to nuclear and tissue immunofluorescence staining. As shown in Figure 3C,E, TDOs was distributed in Kupffer cells, epithelial cells, and hepatocytes, indicating that upon entering the sinusoidal capillaries of the liver, TDO is taken up and degraded by Kupffer cells, epithelial cells, and hepatocytes, with less distribution in non‐parenchymal hepatic stellate cells (Figure S7). Observations of the kidneys (Figure 3D,F) showed that TDOs reaching the kidneys was primarily distributed in the renal tubules and taken up by renal tubular epithelial cells, indicating that TDOs mainly accumulate in the renal tubules rather than being cleared through glomerular filtration, aligning with our earlier speculation. Based on these findings, we suggest that post intravenous injection, TDOs mainly distribute in the kidneys and liver, undergoing metabolism through renal tubular accumulation and within hepatic Kupffer cells, epithelial cells, and hepatocytes. These findings contrast with literature reports indicating the rapid metabolism of structures such as tFNAs within the kidneys,^47^ which implies a comparatively lower metabolic rate of TDOs in the kidneys and a more substantial metabolism occurring in the liver.

### 3.3 Biocompatibility of TDOs

**FIGURE 4:**
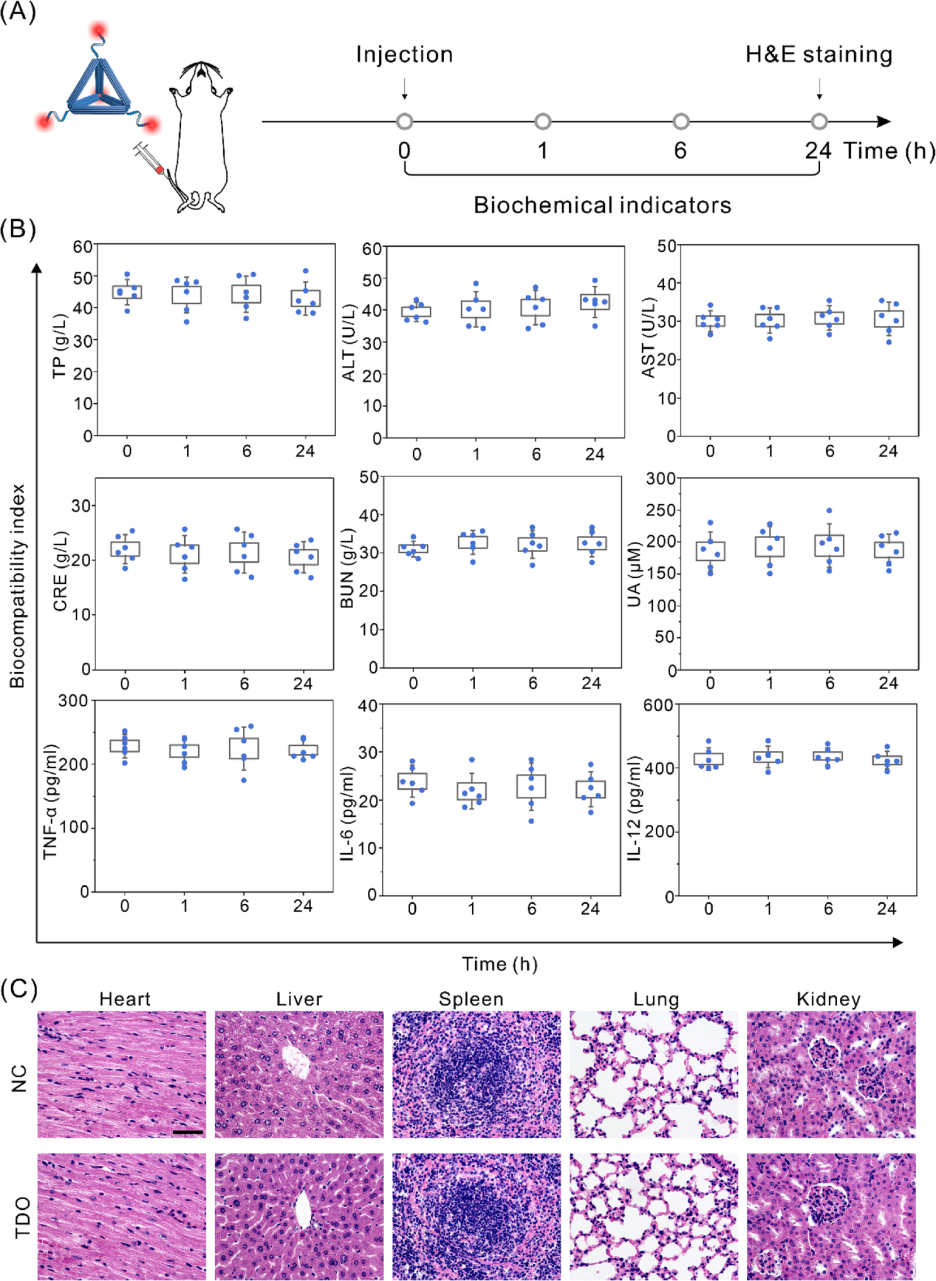
In vivo biocompatibility of TDOs. (A) Experimental design of the biocompatibility assessment. (B) Analysis of biochemical parameters in serum. (C) H&E histopathological sections of heart, liver, spleen, lung, and kidney were analysed at 24 h after injection of saline and TDOs (scale bar: 200 μm).

After elucidating the cellular metabolic pathways of TDOs in the liver and kidneys, various serum biochemical markers associated with hepatic and renal injury were measured, including total protein (TP), alanine aminotransferase (ALT), aspartate transaminase (AST), creatinine (CRE), blood urea nitrogen (BUN), and uric acid (UA) (Figure 4A). Our results did not indicate any significant toxicity of TDOs to the liver and kidneys. Additionally, the immunogenicity of TDOs, an essential factor in assessing the biocompatibility of nanomaterials,^48^ was evaluated by analysing cytokine levels in mouse serum using ELISA, focusing on TNF‐α, IL‐6, and IL‐12.^49^ Following intravenous injection of TDOs at various time intervals (0, 1, 6, and 24 h), no significant changes in TNF‐α, IL‐6, and IL‐12 were observed (Figure 4B), suggesting low immunogenicity of TDOs. Further in vivo biocompatibility tests were conducted through the examination of biopsied tissue samples from the heart, liver, spleen, lung, and kidney using Haematoxylin and Eosin (H&E) staining (Figure 4C). Notably, no evident tissue damage was observed in these stained samples, reinforcing the high biocompatibility of TDOs. Collectively, these findings underscore the potential of TDOs as a promising candidate for drug delivery applications, given its high biocompatibility and low immunogenicity.

### 3.4 Tumour targeting and penetration of TDOs

**FIGURE 5:**
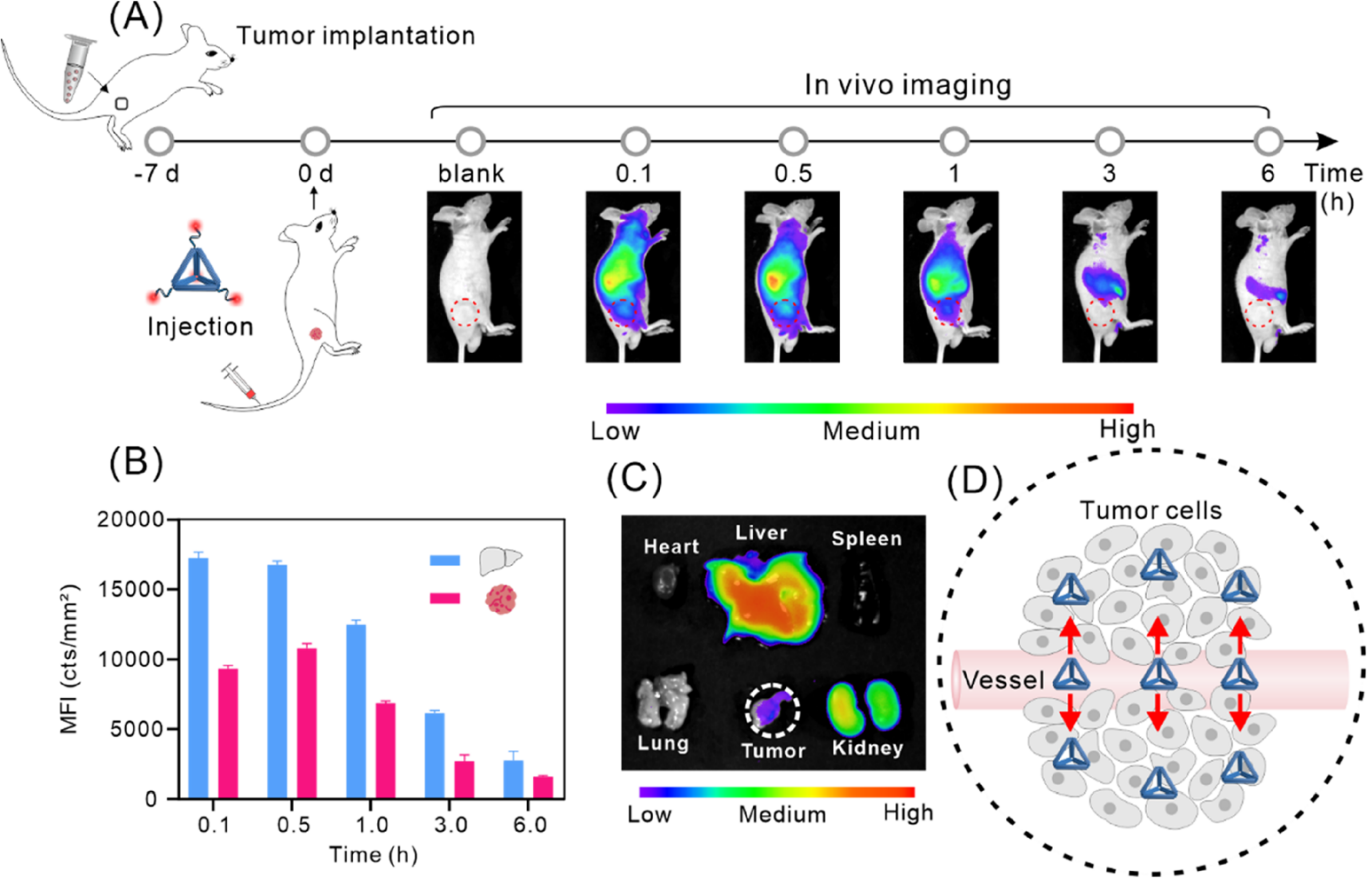
In vivo tumour targeting of TDOs. (A) In vivo imaging of tumour‐bearing mice following tail vein injection of Cy5‐TDOs. (B) Fluorescence quantification of Cy5‐TDOs accumulated in the liver and tumour of tumour‐bearing mice. (C) Ex vivo fluorescence images of major organs and tumour dissected from mice euthanized 0.5 h post‐injection. (D) Schematic illustration of the passive tumour targeting of TDOs.

After demonstrating the metabolism of TDOs in the liver's Kupffer cells, epithelial cells, and hepatocytes, as well as the good compatibility of TDOs, we examined the tumour passive targeting ability of TDOs. We established a subcutaneous tumour model in BALB/c nude mice using HepG2 cells to investigate the tumour‐targeting effect of TDOs following intravenous injection. Figure 5A demonstrates a considerable accumulation of fluorescence intensity at the tumour site, observed at 0.1 h after intravenous injection. The fluorescence reached its peak intensity at 0.5 h and gradually declined thereafter. These findings indicate that TDOs rapidly accumulates in the tumour and is then quickly cleared. Quantitative analysis showed that the peak fluorescence intensity in the tumour was 65% of that in the liver at corresponding times, evidencing significant accumulation of TDOs in the tumour (Figure 5B). Tumours and major organs (liver, kidneys, spleen, lung, heart) were collected 0.5 h after injection for ex vivo fluorescence imaging (Figure 5C). The results showed that TDOs was primarily distributed in the tumour, liver, and kidneys. Moreover, TDOs exhibited a significant tumour fluorescence signal at the tumour site, consistent with the in vivo imaging and quantitative analysis results, thereby confirming its favourable tumour passive targeting capability (Figure 5D).

**FIGURE 6:**
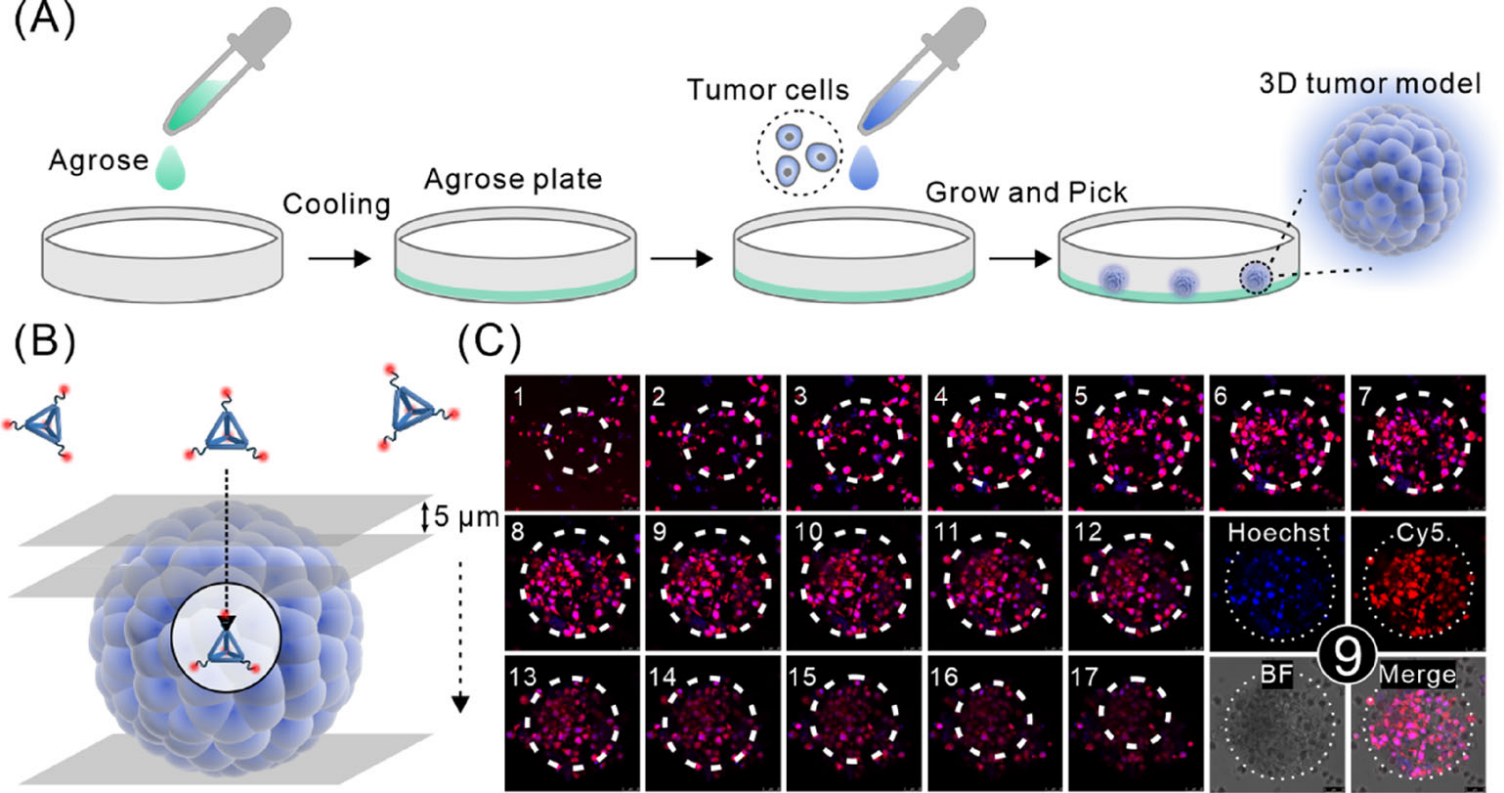
Penetration ability of TDOs in a 3D spheroid tumour model. (A) Schematic illustration of the construction of the 3D spheroid tumour model. (B) Schematic illustration of TDOs penetration in the 3D spheroid tumour model and CLSM Z‐stack scanning (each layer 5 μm thick). (C) Fluorescence images of each layer of the 3D spheroid tumour model, with cell nuclei stained using Hoechst 33258.

It has been demonstrated that the requirement for drug carriers to effectively reach the hypoxic zones within tumours, where proliferating cells reside, is important for exerting therapeutic effects successfully.^50^ The spheroids of 3D tumour model retain the characteristics of tumours in human bodies well, thus regarded as a bridge between cell monolayers and animal models. It is commonly employed to investigate the tumour penetration capabilities of nanomaterials.^51^, ^52^, ^53^, ^54^, ^55^ To determine the ability of TDOs to penetrate the tumour interior, we constructed a 3D spheroid tumour model to mimic the structure of in vivo tumour tissues.^56^ After obtaining the 3D spheroid tumour model using agarose gel‐containing culture dishes (Figure 6A), we incubated Cy5‐TDOs with the 3D spheroid tumour model for 4 h. Subsequently, we stained the cell nuclei and employed confocal laser scanning microscopy (CLSM) Z‐stack scanning techniques to assess the distribution and penetration of TDOs across different layers of the 3D spheroid tumour model (Figure 6B). The results indicated successful penetration of TDOs into each layer of the tumour model, with strong fluorescence signals observed even at the central region of the tumour model (Z = 45 μm) (Figure 6C). This indicates that TDOs possesses high tumour penetrability, as it can reach the central regions of the 3D spheroid tumour model. These findings are consistent with literature reports of 40 nm‐sized nanoparticles showing enhanced penetration and accumulation in tumours.^57^

## 4 CONCLUSIONS

In this study, we have synthesized and characterized TDOs and explored its interactions with various types of cells within the liver and kidneys. We observed that a small quantity of TDOs accumulates in the renal tubules of the kidneys, while a significant amount is metabolized in the liver through the cooperative action of Kupffer cells, epithelial cells, and hepatocytes. After undergoing ‘first‐pass metabolism’ by various liver cells, TDOs demonstrates excellent passive tumour‐targeting capabilities. Furthermore, we demonstrated its ability to penetrate and persist within 3D spheroid tumour model. We hypothesize that the tetrahedral shape of TDOs, providing ‘angular attack’ capabilities, facilitates enhanced interactions with tumour cells, promoting uptake and achieving tumour‐targeting effects. Moreover, its relatively optimal size (40 nm) allows for better penetration and longer retention within tumour tissues.

Previous studies have shown that small‐sized DNA nanostructures like tFNAs are rapidly cleared from the kidneys due to the presence of the slit diaphragm between podocytes in the glomeruli,^47^, ^58^ hindering passive tumour targeting. While targeted delivery to tumours can be achieved by attaching targeting molecules,^59^ this approach introduces complexities in material preparation. Studies have indicated that among nanoparticles of various sizes (40, 70, 110, 150 nm), nanoparticles with size of 40 nm exhibit superior tumour penetration and tissue accumulation.^51^, ^57^ In our study, we chose TDOs with an average particle size of around 40 nm, revealing their excellent tumour penetration and retention capabilities. Considering these factors, we suggest that DNA nanostructures with size of around 40 nm, combining minimal renal metabolism with enhanced tumour penetration and accumulation, are optimal for achieving effective tumour passive targeting. Furthermore, the TDOs structure, with its multiple protruding sticky ends, can be modified with actively targeting molecules like HApt^60^ and AS1411^61^ to further enhance tumour targeting. Various targeting molecules can be attached by utilizing multiple sticky ends in TDOs, providing multitargeting potential and potent cytotoxicity against tumour cells.^62^

Looking ahead, TDOs holds promising prospects due to its multiple modification sites, well‐defined metabolic pathways in liver and kidney cells, and tumour‐targeting characteristics. These features allow for the rational functionalization of TDOs with active targeting elements and controlled release components, facilitating precise and controllable cellular metabolism regulation and targeted tumour diagnostics and therapy. Additionally, the large internal cavity volume and stability of TDO make it suitable for loading various drugs into the central cavity, opening avenues for combination or cocktail therapy. Finally, TDOs' capability to construct superlattice devices positions it as an intelligent drug delivery system. Consequently, TDOs represents a new generation of DNA nanocarriers for drug delivery, advancing the biomedical and therapeutic applications of nucleic acids frameworks.

## AUTHOR CONTRIBUTIONS

Shitai Zhu and Hongzhen Peng performed the experiments. Qinglong Yan helped in guidance of imaging experiments. Kai Xia helped in guidance of TDO preparation. Ying Zhu and Lihua Wang performed critical revisions. Shitai Zhu, Ying Zhu, Huating Kong, and Shihua Luo wrote versions of manuscript. All authors discussed and commented on the manuscript.

## FUNDING INFORMATION

This work was supported by the National Key R&D Program of China (2023YFC3404200), the National Natural Science Foundation of China (22022410, 82050005, 12005281, 21874043, 12305400 and 22274097), the Shanghai Municipal Science and Technology Major Project (19142202400, 19JC1410300, 2018SHZDZX03), 2022 Shanghai ‘Science and Technology Innovation Action Plan’ Fundamental Research Project (22JC1401203), the Science Foundation of the Shanghai Municipal Science and Technology Commission (21dz2210100), the Shanghai Science and Technology Development Funds (22QB1404000) and the Open Research Fund of the National Facility for Translational Medicine (Shanghai) (TMSK‐2021‐412). This work was also supported by the User Experiment Assist System of Shanghai Synchrotron Radiation Facility (SSRF).

## CONFLICT OF INTEREST STATEMENT

The authors declare no conflict of interest.

## Supporting information


**Data S1.** supporting Information.

## Data Availability

The data that support the findings of this study are available from the corresponding author upon reasonable request.
