# Supplementary material for: Visualization of the hepatic and renal cell uptake and trafficking of tetrahedral DNA origami in tumour
Source: Cell Prolif. 2024 Apr 4;57(8):e13643. doi: 10.1111/cpr.13643 (PMC11294413; doi:10.1111/cpr.13643)
Supplement: Supplementary file 1 — Data S1. supporting Information. [file CPR-57-e13643-s001.docx]

**Supporting Information**

Visualization of the Hepatic and Renal Cell Uptake and Trafficking of Tetrahedral DNA Origami in Tumor

Shitai Zhu^1,7#^, Hongzhen Peng^2,3#^, Huating Kong^2^*, Qinglong Yan^3,5^, Kai Xia^4,5^, Lihua Wang^1,3,7^, Ying Zhu^1,3,7^, Shihua Luo^6^*

^1^ Division of Physical Biology, CAS Key Laboratory of Interfacial Physics and Technology, Shanghai Institute of Applied Physics, Chinese Academy of Sciences, Shanghai 201800, China

^2^ Shanghai Synchrotron Radiation Facility, Shanghai Advanced Research Institute, Chinese Academy of Sciences, Shanghai 201204, China

^3^ Institute of Materiobiology, College of Science, Shanghai University, Shanghai 200444, China

^4^ Shanghai Frontier Innovation Research Institute, Shanghai 201108, China

^5^ Xiangfu Laboratory, Jiashan 314102, China

^6^ Department of Traumatology, Rui Jin Hospital, School of Medicine, Shanghai Jiao Tong University, Shanghai 200025, China

^7^ University of Chinese Academy of Sciences, Beijing 100049, China

^#^ Shitai Zhu and Hongzhen Peng contributed equally to this study.

**Corresponding Authors**

* E-mail: konght@sari.ac.cn

* E-mail: jqab@163.com


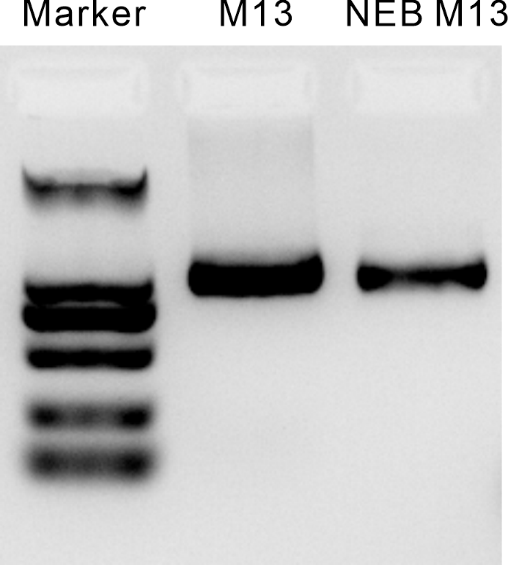


**Figure S1.** 1% agarose gel electrophoresis analysis of M13 scaffold strands. From left to right: DL2000 DNA marker, self-extracted M13 scaffold strand, and commercially available scaffold strand.


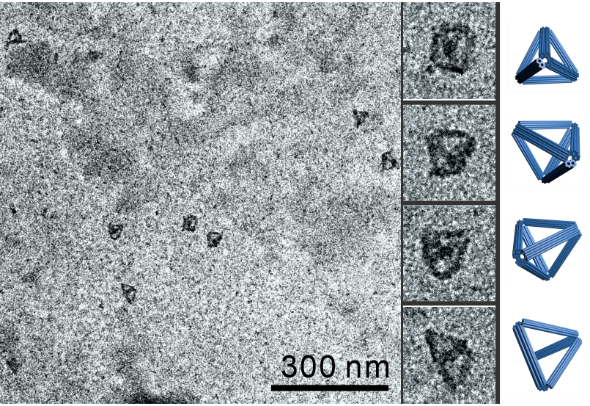


**Figure S2.** Transmission Electron Microscopy (TEM) images of TDO (scale bar: 300 nm).


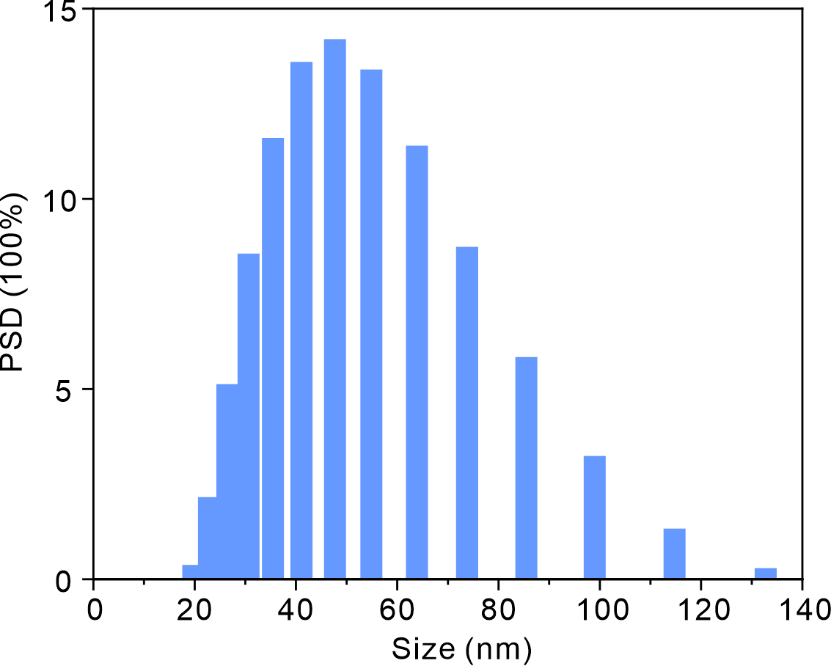


**Figure S3.** DLS measurement statistics of Cy5-TDOs.


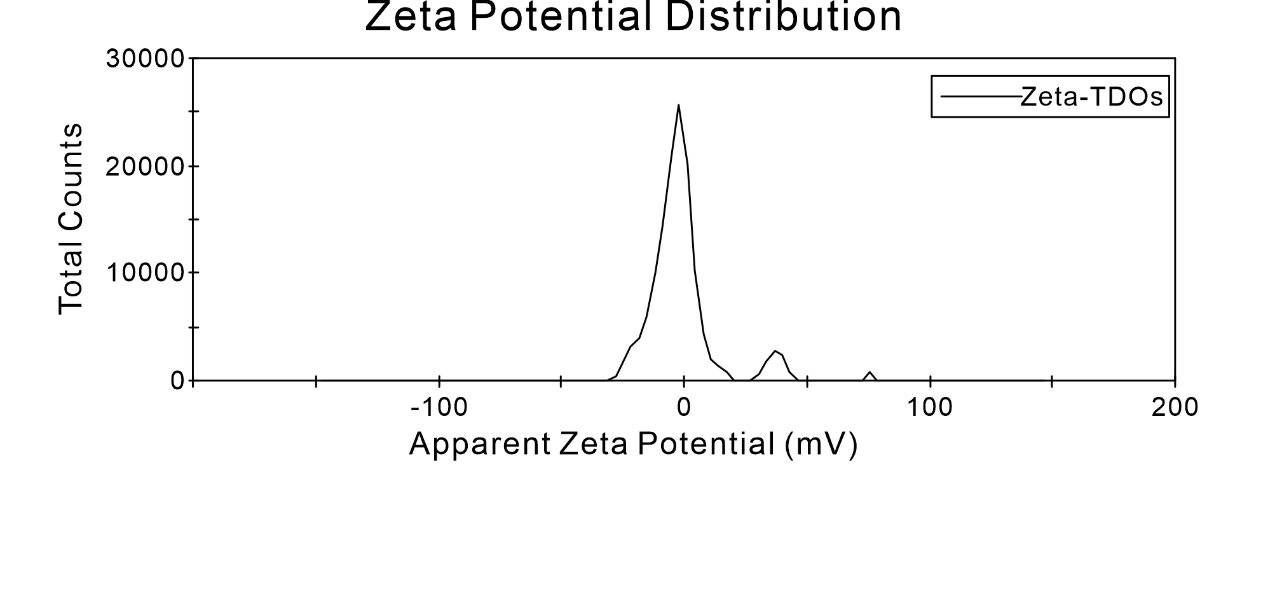


**Figure S4.** Zeta potential measurement of TDOs.


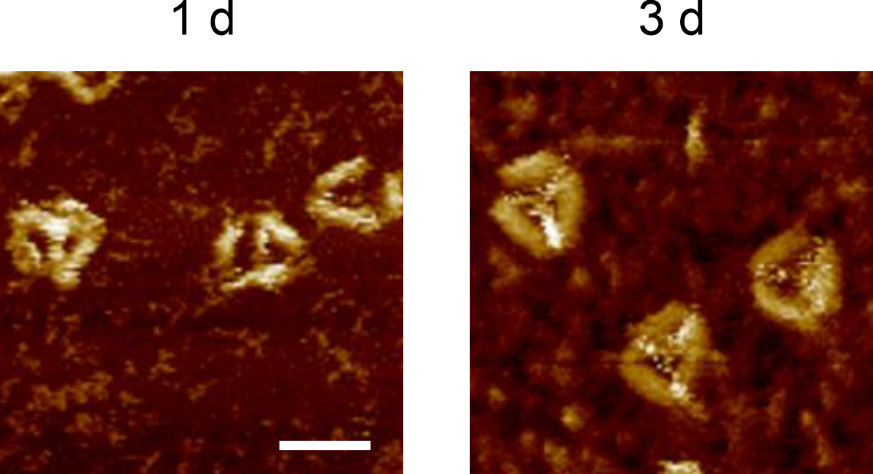


**Figure S5.** AFM images of TDOs stored in TE/Mg^2+^ buffer for 1 day and 3 days (scale bar: 50 nm).

**
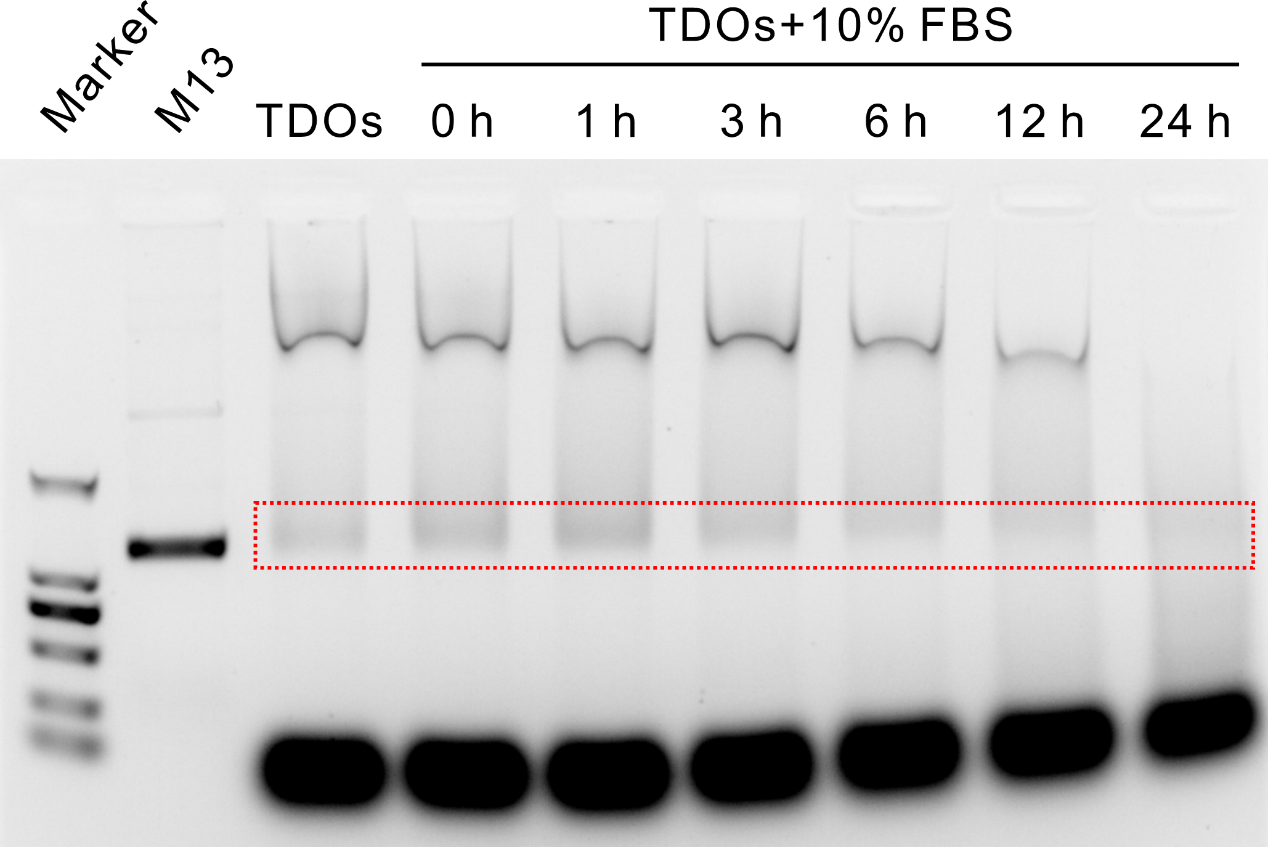
**

**Figure S6.** Agarose gel electrophoresis images of TDOs incubated with FBS, marker: DL2000.


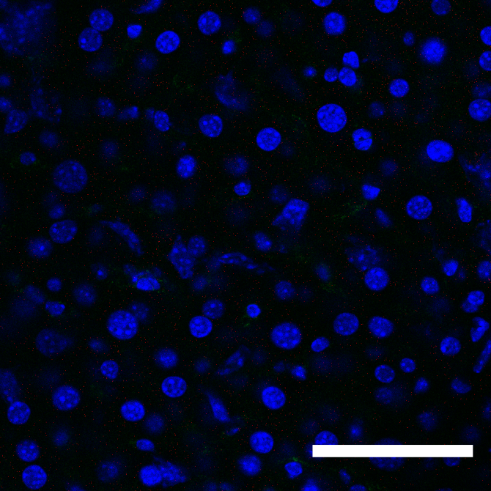


**Figure S7.** Confocal observation of distribution of TDOs in the liver, with hepatic stellate cells marked by Desmin (scale bar: 50 μm).

**Table S1.** Sequences of staple strands used in this work.

| TO1 | ATTGTGTAGCCGTCCCCGAACATACCGAACGAACCCAGC |
| --- | --- |
| TO2 | ATTTGTATTGAGGACAACTCGGAAGATA |
| TO3 | GCGCGAAACAAAGTACCCAGCGATTATACCAA |
| TO4 | ACCGAACTGACCAACTGGTCAATCATAAGGGA |
| TO5 | AGGACAGATCTTGAGAATAACATAAAAAAACACCCGAAT |
| TO6 | ACGTAGCCGGAACGAGGCCCACAATGAACAATTTAATG |
| TO7 | CAGGCGCATACCGCGACCTGCTCCACCAATAAGGGAGAACCTAAAA |
| TO8 | CTCATCTAAAATACAAAGAAACCACCAGAAGGAGCGGAATT |
| TO9 | GCTGACGCGCATTAGAAAAGTTTGAGTCAACCTAAAAGGCTG |
| TO10 | AGGAAGTTTCCATTAAACGGGTTTGACCCAACGGAG |
| TO11 | AAAATGAAAATAGCAGCCTTTGGGTAATTGAGC |
| TO12 | GGAACTTCATCAAGAGTAATGA |
| TO13 | CATTTTTCCTTTGAATAGATTAAAATATCTTTAGTGAACCTTGAAAAA |
| TO14 | AACGTAATGCCACTACGAATACACCTGATAA |
| TO15 | TTTACAAACAATTCGATTTAGAAGTATTAGAC |
| TO16 | GCTAATATCAGACGAAGCCCTTTTTAAGAAA |
| TO17 | TGAAAATAGCAATAGCTCCAGAAGAAGACTC |
| TO18 | GATTAGCGAAATCGAAAGAGGCAAAAGAAGGCACAACATTAT |
| TO19 | ATGGTTAAGCTGTTACTGTGTACAGAC |
| TO20 | ATCAACAGTTGAAAGGAATTGAGGAATCAA |
| TO21 | AGTAAGCAGATAGCCGAACAAAGTTAATCTTACGAGATAACGCAGACTTGAAAG |
| TO22 | ATAACGTCACCTTGCGAGCACTAACAAAGAGCAAGAGCAATA |
| TO23 | TCAACGCTGAGAGCCAGACCAGCATATTAAAGCGG |
| TO24 | CTTATTAATTAGTCAGTCAGAACAGAGACAAGAACCGGATATTCATTACCCAA |
| TO25 | TCTAAAGATTAAAAGTTATTAATTTTACTACTAATA |
| TO26 | AAACAAATATCAAACCCGGTTATCAATACATTCATCGCTAAAACA |
| TO27 | GACAATATTTTTGAATGGCTCGCAGTATGTTAGCAAACG |
| TO28 | CGCGAACCATGATTGAAACCGAGGAAACAACA |
| TO29 | CGTAATGAACTCAATATTTTCATCGCCCAGAATACCCAAAAGAACTGGTGATAGCTTAACTGCAG |
| TO30 | AAACAGACAGTGCCATATCTGGTCAGTTGGCAA |
| TO31 | TAATTTGATTTACGACTCATCCGCCGCCAGCATTGGAAA |
| TO32 | AACGAGCAATCAATGGGTATTGTTGAGG |
| TO33 | TCAACGTAACAAAGCTGCACGATTTTTTGTTTAACGT |
| TO34 | GTGAATAGGTTTAATTATACCAGTCAGGGAACTAACCAC |
| TO35 | TTGTTATCCCAATCCAAAGGAATACGGAACAAGCCACC |
| TO36 | CGTAATGAACTCAATATTTTAACAGCCTGCAGATTACGTTACACCAGA |
| TO37 | TTAGTTGTCTAAGAGCGCCCAATAGCAAGCAAATCAGATA |
| TO38 | GCACCCAGCTACAATTTTTTTCGCTGAGGCTTGCAGGGAGTCATCGCCCACGCATAACCGATTTTTTTATCCTGAATCTTAC |
| TO39 | TAGTAAGAAGAAAAAGCCGTTTTTATTCTCCCGACTAACGAG |
| TO40 | AGAAGGCTTATCCGGTATCTATTTTCAACGCT |
| TO41 | ATGCGATTTTAAGAACTACAGGTAGAAA |
| TO42 | TTGGATTGGGCTTGAGATAGGC |
| TO43 | AGGAATGTACCGCAGCATGTAGATAAGTCCTGAAAAGCGTCCAGTCTC |
| TO44 | ACCACGCGAGGCGTTTTGCCTTAACAGAGCC |
| TO45 | TTATCATTCCAAGAACAATCGGCTGTCTTTCC |
| TO46 | GATTCATCAGTTACACTATCATAACCCTCGTT |
| TO47 | ATTTTACGAGGCATAGTATAGCCCACCACCG |
| TO48 | ATCCTACCAGTTACGGGAGGTTTTGAAAGCGAACTTCATCGT |
| TO49 | GAACAACTAAATATTATCCCTGACGAG |
| TO50 | TGTTCAGCTAATGCAGAACGCGCCTGTTTATATGA |
| TO51 | GCGTTTTCATCGGCATTTTCGGTCAAGAGCAGAGATTTATAAGAATCATTCA |
| TO52 | TTTTCACGTTCCAGTCAAGAAAAATAAATAACGCCAGCCATC |
| TO53 | TACTTAAAGCCAGAATGACAGGAGAAACCAACATT |
| TO54 | GAACCGCCCCTCAGACATTATTGGCTCATTTCAACTTTAATCATTGTGAATTAC |
| TO55 | TGAATTTCCAGAGCGAGAACAAGCAATCACTATCCC |
| TO56 | GCGATACATGGCTTTTGCAACAATAGAAACCGTCTTTCATCAAGA |
| TO57 | ATTCACAAACAAATAAGACGATTGGCCTTGAT |
| TO58 | ACCCTCAGAACCGCCACTCCCTCAGAGCCGCC |
| TO59 | ACCCTCACAGAGCCCCTTATTAGCGTTTAAAG |
| TO60 | ACCACCAACTAATCAAAATCACCGGAACGAGCCGCATAAAACACG |
| TO61 | CAGGTCAATCCTCAAGGAGTGTACTGGTAATAAGTTT |
| TO62 | GAATGACATCACCGAACGTCATCTGGCCAACAGAGAAAC |
| TO63 | TTTAAACCATTTGGATTACCACCTTCTG |
| TO64 | CATTGAATCCCCCTGGAATCGTCATAAATA |
| TO65 | AAGATTAAGAGGAAGCAGCGGATTGCATCAAA |
| TO66 | ACTTCAAAACAGGTGGATGGCTTAGAGCTGTTTTATTCC |
| TO67 | CGCGACTATTATAGTCACAGTTGAAATATGCTGGCAGA |
| TO68 | GAGCTTCAAGGCAAAAATCAGGTCTAACGAGTGCTGTAGGACCAGT |
| TO69 | GCGTCCAATAGCGATCAAGTTTGCCTTTAGCGTCAGACTGT |
| TO70 | AGAGTACCTTTAATTGCTTTTGAATCGCCATATTTAACAACGGCCAA |
| TO71 | CCAGACCTGAATATATCGATAGCAGCATTGCCAGAGAGCGAA |
| TO72 | CAGACGACGATAAAAACCAAAATACTGCCAAATGC |
| TO73 | ATTGCGGAAGCAAACTCCATAT |
| TO74 | AGTAGCGGCCGGATCACCGAGGTAAATATTGACGTGTCACAAAGACAC |
| TO75 | GAAGAGGCTTTTGCAAAGTTTAGAAAAACGA |
| TO76 | AATCACCAGTAGCACCGAATTAGAGCCAGCAA |
| TO77 | GAAGTTTCATTCAATAACCTGTTTAGCTATA |
| TO78 | CAAAGATACATTTCGCAGGTGGCAGCAAAGA |
| TO79 | TGAATTCATAAATGGTAATAGTAAAATAGAAGTTCCGTAATC |
| TO80 | ATTTTCTGCGTTACCCTGTTTTAATTC |
| TO81 | AAAGGGCGACATTCAACCGATTGAGGCATA |
| TO82 | CATTTGGGGCGCGAGCTGAAAAAATGGTCCATATAAGAAGCAACCGAAAG |
| TO83 | AGCATTAGTTTATTTGAAATTATTCATATTTAGTTTAGTAGT |
| TO84 | TGGGCAACATATAAAAGATAGAACTTAGCAAGACA |
| TO85 | ATTAGCATTTACATAACTAAATTTTTGCCAGGATTAG |
| TO86 | CACGGAAGGGACATCCAATGAAACCAATAGTAAAGG |
| TO87 | GCAATCAATAGAAAATTGAGGGAACTTGAGCAGTTCAGCTGGATA |
| TO88 | AGAAAATACATACATAGCGTAAGAATACGTGGCA |
| TO89 | CAATCGTCTGAAATGGATTAAAATTAAGCAATAAAGCCT |
| TO90 | TTCACCAAGGCAAGTCAATTCTACTAATGACC |
| TO91 | CGTAATGAACTCAATATTTTAATAAAATAAACATCCAATAAATCATACGTCACACCTCAACATTA |
| TO92 | ACCTGAAAAAGGTGTTTACCAGCGCCAAAGACA |
| TO93 | CACGACGTTACATCTTTCAATCGGAACGAGGGTAGCAAT |
| TO94 | TAACGCCCGGATTCCGCGCAGTACAGAG |
| TO95 | GCTGCAAGGCGATTATGGCGAAAGGGGGATG |
| TO96 | CCGAGCTCGAATTCGTAGAGGATCCCCGGGTA |
| TO97 | GTCATAGCCGGAAGGCCCTCATAGTTAGACGTTAGGGAG |
| TO98 | TTCGCATGCCTGCAGGTTTTCAGCTAAATGACGCTTTT |
| TO99 | TTGTTATCCTTACGACGGCCAGTGCAAGGAACTGTCGTCCTCAGCA |
| TO100 | GCTATTAAGGCAAAATTAATTACATTTAACAATTTCATTTGAATT |
| TO101 | AATTCCATCTAAAGTAGAAGATGATGAGCGCAACTGGCTCAC |
| TO102 | ACCGCTTCTGGTGCCGGAAACCCGCCAGCAGTTGGG |
| TO103 | ACTACAACGCCTGTAGCATTCCGTATGGGATTTT |
| TO104 | ACGACACAACATACGAGCTGT |
| TO105 | TCAAGATTATTCAGGGAGAAAGGTTTAACGTCAGTGAATAAATCCTGA |
| TO106 | AAAGCGCCATTCGCCATGTGCGGGTCCCAGT |
| TO107 | TACCAAGTTACAAAATGCCTGATTGCTTTGAA |
| TO108 | GCTAAACAACTTAATCTCCAAAAAAAAGGCT |
| TO109 | TGATAATAATTTTTTCATTTATCAACAATGA |
| TO110 | TACCTTTTGTAAAGGGAAGGGCGATCGTCAGGCTAACAAACA |
| TO111 | GAAGAATAGACAAGCTTCTGTGTGAAA |
| TO112 | AAAACAGAAATAAAGAAATTGCGTACCTA |
| TO113 | CCAAAAGGAGCCTTTAATTGTATCGGCGTTGAATCAACAGCGACTCTAATCATG |
| TO114 | TCTTAATTATACTTCATGAATATACAGCTAAAGGAATGAATT |
| TO115 | CCATGATGGCAATTCATCAACGGCAGGCGAAAAAC |
| TO116 | CAACAACTAAAGGCATTTTCTACAGACACATAAAGTGTAAAGCCTGGGGTGCC |
| TO117 | TTGTTTGACAGCATTACCTGAGCAAATTAATAACAG |
| TO118 | ATATGGAAGGGTTAGAAGATTTTCACAATAAAGGGTTTCCTCTTC |
| TO119 | ATCATATTCCTGATGGACTAAAGACTTTTTC |
| TO120 | GCGGGATTGCGCCGGCTTGCTTTCGAGGTTGC |
| TO121 | CGTAATGAACTCAATATTTTGCGAAAGGAACAGCTTGATACCGATAGTCGTCACCTTTCCAGCGTA |
| TO122 | GCTTTGATATCAGATATCAAAATTATTTGCACG |
| TO123 | ACAGCTGATAGCCCCTCAGAGATAAAGTACCGACAAACA |
| TO124 | TTTTCACACCGTACGCCACCCAAGTAAT |
| TO125 | GGGCGCCAGGGTGGAGAGGCGGTTTGCGT |
| TO126 | GGCGAAAATCCTGTTTCTGGTTTGCCCCAGCA |
| TO127 | GTTCCGAGTTGTTCTCAAAATCATAGGTGTTGGGTCGAG |
| TO128 | GGCAGTTGCAGCAAGCGAAGAACGTATATAAGTAATTT |
| TO129 | ATAAATCAAAATTCACCGCCTGGCCCAAATATAACCTCCTCGAGCC |
| TO130 | GGCCAACAATTGCGGTACCGTAACACTGAGTTTCGTCACCAGTAC |
| TO131 | AGCCCGACTACCTTTTTTTCAGGGATACAGTCGGGAAAGAAT |
| TO132 | AATGAGTGAGCTAACTCACATTGCGCGGGGTTTTTC |
| TO133 | GACGCTGAGAAGAGTCAATAGTTAAATGCTGATG |
| TO134 | GAGAGATAGGGTTGAGTAATC |
| TO135 | AATAGGCGCCACCGGAATAGCCAGGCGGATAAGTTATTATTAATGCCC |
| TO136 | CATTTGCGCTCACTGCCTGCATTAACGGGCA |
| TO137 | CGCCACCCTCAGAACCTCAGGAGGTTTAGTAC |
| TO138 | CAAATCCAATCGTTGAAATACCGACCGTGTG |
| TO139 | AAATTCTGACCTAAATTACACCGGCTTACCA |
| TO140 | ATAAGTATTGCCCCCTGTCGTGCCAGCCGCTTTCGCAAGCCC |
| TO141 | ATCACTTTTTCTGAGAGAAAATCCCTT |
| TO142 | GAAGGATTAGGATTAGCGGGGTTTTGTATT |
| TO143 | ATAAATAAGGCGTTAAATAAGAATAATAATGGTCAAGACAGTCCACGGATGGTG |
| TO144 | GCCTGTTTCGGAACCGCCGTCGAGAGGTTTAGTTAAGAAAAA |
| TO145 | AAGACAGTGCCCGTATAAAAGGTATCAGAACAACC |
| TO146 | CGCTCAACAGTAGGGCAATTTCCTTTTGATAAGAGGTCAGTACGGTGTCTG |
| TO147 | GTATAAACCAACATCTATATGGAATTTACAGTTTGGAACAAGAGTCCACTATT |
| TO148 | CCTGCCTAGAGAATCCACCACCCTCATTATGTTGAT |
| TO149 | GTTCTGAAACATGAAAGCTCAGTAGTGTATCCAGTGAGATGAATC |
| TO150 | AACGGGGTCAGTGCCAGACGACGACAATAAACAA |
| TO151 | AGGCAGAACAAATTAATCATAATTACTATTTC |
| TO152 | CGTAATGAACTCAATATTTTAGTAATAATTTAGTATCATATGCGTTATGGCATTTGGCTTAGCTGA |
| TO153 | TCTGTCCTTGAGTAAGGCTGAGACTCCTCAAGA |
| TO154 | ACCTTGCGCATTAAGCCAGCTAATCACCATCAATACAGG |
| TO155 | ATCAATAAGCTCATTTCGCGTCAACCGT |
| TO156 | CTTTTTTAATGGAAACAGCAGCCAGCTTTCCGG |
| TO157 | AAGAACGTGGACTCCAACCATAGCGATAGCTTAGATT |
| TO158 | GGCGAAAGATTTTACACCGAGTAAAAGAAATACTTCCTT |
| TO159 | GTCTTCCCTTAGAATCCCTATCGGCTTTGATTAGGTAA |
| TO160 | AGAGCGGTTAATCGTCGCTATTATCCAGAATTAACCGGAGAAAG |
| TO161 | GGAAGATGGTGTAGCGTGGGAACAAACGGCGGATTGACCG |
| TO162 | AACAGGCATCACGCAAAATGTGAGCGAATCTGCCAGGAGCTA |
| TO163 | AATGGGATAGGTCACGTTCGCACTCTACATAA |
| TO164 | AGAAGTGTTTTTATAATCAAACATCACTTGC |
| TO165 | TGTCAGGCCGATTAAAGGAACC |
| TO166 | CCGTCGTCCTGTAATTTTTGATATTTAAATTGTAGAGAATCTTGCCTG |
| TO167 | CTCATGGGCGCATCGTACAGTATCGTGAATA |
| TO168 | ACGCCATCAAAAATAATTTTTAACCAATAGGA |
| TO169 | CTGAGTAGAAGATGGAAATACCTACATTTTGACG |
| TO170 | GCTTTGCAACAGGAAAACCAAAAAATAAAAA |
| TO171 | AAATTCTTCTGTATGAGGGGACGACGAACCGTGCGTAACAAC |
| TO172 | CCAGGTAATAATTAATTTATCATAGAATC |
| TO173 | AGAGCATAAAGCTAAATCGGTTGTAACGCTCAACTCAAATTGAAAAGTCAAAG |
| TO174 | TTTGCGAGCAAACAAAACGTTAATATTATATTACCGAATACT |
| TO175 | AACTCTACAAAGGCTATTGATATTCTGGCCTGATT |
| TO176 | TCATATGTACCCCGGGGTTAACACCGCCTGCAAGGTGAGGCGGTCAGTGGCAGAAAAGCCCCAAAAACAGGAAGATTGTAGTAA |
| TO177 | TTTTTAGTAATGTGTAGTAATGTGAGGCGACAGGAACGGTACGCCAGAATCCTG |
| TO178 | AGAGTCTACAGTCATTCATCAACATTAACATTGTTA |
| TO179 | TCAGATGAACGGTAATCTAAGCAATTAAATCTATGTGAGGCCTCA |
| TO180 | AGAGGGTAGCTATTTTGATAAATTAATGCCGG |
| TO181 | AAATGCAATGCCTGAGAACCCTCATATATTTT |
| TO182 | AGATTCACGCAAGGCATTATGACCCTGTCCAG |
| TO183 | GCCGGAGGGGGAGAAGCCTTTATTTCAAAAAGGGTTTGTAGCGTC |
| TO184 | TCTAGCTTGAGAGATAGCATGTCAA |

**Table S2.** Fluorescent strands and arm strands linked to fluorophores

| 5’-Cy5 | Cy5-GTGGTAGTTAGGAGTGAATGC |
| --- | --- |
| arm1 | GCATTCACTCCTAACTACCACTTTTTGCGCGAAACAAAGTACCCAGCGATTATACCAA |
| arm2 | GCATTCACTCCTAACTACCACTTTTTTTTACCGAACTGACCAACTGGTCAATCATAAGGGA |
| arm3 | GCATTCACTCCTAACTACCACTTTTTTTTATCAACAGTTGAAAGGAATTGAGGAATCAA |
| arm4 | GCATTCACTCCTAACTACCACTTTTTAGTAAGCAGATAGCCGAACAAAGTTAATCTTACGAGATAACGCAGACTTGAAAG |
| arm5 | GCATTCACTCCTAACTACCACTTTTTTTTTAGAAGGCTTATCCGGTATCTATTTTCAACGCT |
| arm6 | GCATTCACTCCTAACTACCACTTTTTCTTATGCGATTTTAAGAACTACAGGTAGAAA |
| arm7 | GCATTCACTCCTAACTACCACTTTTTATTCACAAACAAATAAGACGATTGGCCTTGAT |
| arm8 | GCATTCACTCCTAACTACCACTTTTTTTTACCCTCAGAACCGCCACTCCCTCAGAGCCGCC |
| arm9 | GCATTCACTCCTAACTACCACTTTTTTTCATTGAATCCCCCTGGAATCGTCATAAATA |
| arm10 | GCATTCACTCCTAACTACCACTTTTTTTTAAGATTAAGAGGAAGCAGCGGATTGCATCAAA |
| arm11 | GCATTCACTCCTAACTACCACTTTTTTTTAAAGGGCGACATTCAACCGATTGAGGCATA |
| arm12 | GCATTCACTCCTAACTACCACTTTTTTTTTCATTTGGGGCGCGAGCTGAAAAAATGGTCCATATAAGAAGCAACCGAAAG |
| arm13 | GCATTCACTCCTAACTACCACTTTTTTGCTGCAAGGCGATTATGGCGAAAGGGGGATG |
| arm14 | GCATTCACTCCTAACTACCACTTTTTTTTCCGAGCTCGAATTCGTAGAGGATCCCCGGGTA |
| arm15 | GCATTCACTCCTAACTACCACTTTTTTTTTAAAACAGAAATAAAGAAATTGCGTACCTA |
| arm16 | GCATTCACTCCTAACTACCACTTTTTCCAAAAGGAGCCTTTAATTGTATCGGCGTTGAATCAACAGCGACTCTAATCATG |
| arm17 | GCATTCACTCCTAACTACCACTTTTTATTGGGCGCCAGGGTGGAGAGGCGGTTTGCGT |
| arm18 | GCATTCACTCCTAACTACCACTTTTTTTTGGCGAAAATCCTGTTTCTGGTTTGCCCCAGCA |
| arm19 | GCATTCACTCCTAACTACCACTTTTTTTTGAAGGATTAGGATTAGCGGGGTTTTGTATT |
| arm20 | GCATTCACTCCTAACTACCACTTTTTATAAATAAGGCGTTAAATAAGAATAATAATGGTCAAGACAGTCCACGGATGGTG |
| arm21 | GCATTCACTCCTAACTACCACTTTTTTTTTAATGGGATAGGTCACGTTCGCACTCTACATAA |
| arm22 | GCATTCACTCCTAACTACCACTTTTTAGAAGTGTTTTTATAATCAAACATCACTTGC |
| arm23 | GCATTCACTCCTAACTACCACTTTTTAGAGGGTAGCTATTTTGATAAATTAATGCCGG |
| arm24 | GCATTCACTCCTAACTACCACTTTTTTTTAAATGCAATGCCTGAGAACCCTCATATATTTT |
